# Comparison of perineural and systemic dexamethasone use in impacted third molar surgeries in terms of anesthesia duration and postoperative complaints: a controlled, randomized observational study

**DOI:** 10.1186/s12903-024-04483-4

**Published:** 2024-06-18

**Authors:** Doğan Ilgaz Kaya, Ahmet Aktı

**Affiliations:** 1 Faculty of Dentistry, Oral and Maxillofacial Surgery, Karamanoglu Mehmetbey University, Karaman, 70200 Turkey; 2 Faculty of Dentistry, Oral and Maxillofacial Surgery, Selçuk University University, Konya, 42130 Turkey

**Keywords:** Anesthesia duration, Impacted third molar, Dexamethasone, Perineural

## Abstract

**Background:**

Surgical extraction of impacted third molars (ITM) often leads to postoperative discomfort including pain, swelling, and limited function. Steroids like dexamethasone (DXN) are commonly used in oral surgery to manage pain and inflammation. Various administration routes for DXN exist, including intravenous (IV), perineural (PN), and oral applications, each with its advantages. Previous studies have shown that adding DXN to local anesthetics can prolong anesthesia duration and reduce postoperative sequelae. However, comparative studies on IV and PN applications with inferior alveolar nerve block (IANB) of DXN in ITM surgeries are limited.

**Methods:**

This controlled, randomized observational study involved patients undergoing Class II position B ITM extraction. Patients were divided into three groups. IANB (1.8 ml of articaine hydrochloride + 1 ml of saline) was performed 1 h after IV-DXN (4 mg/ml DXN) was administered to the IV group. DXN along with IANB (1.8 ml of articaine hydrochloride + 1 ml of 4 mg/ml DXN) was applied to the PN group. Only IANB (1.8 ml of articaine hydrochloride + 1 ml of saline) was applied to the control group. Anesthesia duration was assessed as primary outcomes. Anesthesia duration was evaluated using a vitalometer from the molars. Secondary outcomes included postoperative pain and edema measured on the 1st, 3rd, and 7th days after surgery. Pain was evaluated postoperatively by using a visual analog scale. A *p*-value < 0.05 was considered statistically significant.

**Results:**

The study included 45 patients with similar demographic characteristics across groups. IV application significantly prolonged anesthesia duration compared to the control group. (*p* = 0.049) Both IV and PN administration of DXN reduced postoperative edema at 3rd (*p* = 0.048) and 7th day (*p* = 0.01). Post-procedure pain reduction was significant in the IV group (*p* = 0.011). On the other hand, it was observed that the pain did not decrease in the PN group at 3rd and 7th days compared to the control and IV groups.

**Conclusions:**

PN and IV DXN administration prolonged anesthesia duration and reduced postoperative edema in ITM surgeries. However, PN DXN administration was associated with increased postoperative pain compared to IV DXN and control groups. Further studies comparing different doses and administration routes of DXN are needed to determine optimal strategies for managing postoperative discomfort in ITM surgeries.

**Trial registration:**

This study was conducted at Ahmet Keleşoğlu Faculty of Dentistry with the permission of Karamanoğlu Mehmetbey University Faculty of Medicine Ethics Committee (#04-2022/101). Trial registration is also available at clinicaltrail.gov. (NCT06318013, 26/05/2024)

## Introduction

Although surgical extraction of impacted third molar (ITM) is a routine procedure for surgeons, postoperative pain, swelling and limited chewing function are difficult for patients. The discomfort that occurs after the procedure may vary depending on the difficulty of the procedure, the preferred surgical technique, and how deeply impacted the extracted tooth is [1, 2]. The use of steroids is frequently preferred in oral surgery to control pain and reduce the body’s inflammatory response [3, 4]. 

Dexamethasone (DXN) is a synthetic analog of prednisolone. It is used in oral surgeries due to its strong anti-inflammatory effects. For steroid administration before or after ITM surgery, routes of use, such as intravenous (IV), intramuscular, submucous, perineural (PN), oral or perioral applications, are available in the literature [4–6]. These routes may vary depending on the physicians’ experience.

One of the anesthesia solutions frequently used in ITM surgeries is articaine. The high success rate, providing anesthesia in a short time, good control of pain during and after the procedure, and long duration of anesthesia play a role in the preference for articaine. In their animal studies, Castillo et al. showed that PN administration of DXN along with the anesthetic solution prolongs the duration of anesthesia [7]. Kopacz et al. demonstrated that anesthesia and analgesia can be increased for up to 96 h by adding DXN during intercostal anesthesia in human volunteers [8]. 

In literature research, it has been found that by adding DXN to lignocaine and ropivacaine during ITM surgery, anesthesia duration was increased and postoperative sequelae were less in PN applications [6, 9]. In another study, it was shown that anesthesia duration increased equally in IV and PN DXN applications during thoracic paravertebral block [10]. 

The hypothesis of this study was that DXN, which is frequently used in oral surgery, would similarly prolong the duration of anesthesia with articaine and improve postoperative sequelae in PN and IV patients.

Therefore, the aim of this study was to investigate the effect of adding DXN to articaine solution simultaneously with inferior alveolar nerve block (IANB) and administering DXN IV before the procedure on the duration of anesthesia, pain and swelling after the procedure.

## Methods

This study was conducted at Ahmet Keleşoğlu Faculty of Dentistry with the permission of Karamanoğlu Mehmetbey University Faculty of Medicine Ethics Committee (#04-2022/101). Trial registration is also available at clinicaltrail.gov. (NCT06318013, 26/05/2024) This study was conducted in accordance with the 1975 Helsinki Declaration, as revised in 2013 also written in accordance with CONSORT guidelines. In this study, patients who underwent Class II position B ITM extraction according to the Pell and Gregory classification were observed.

In the present study, patients who made an appointment at the department of maxillofacial surgery for ITM extraction were observed. ASA I nonsmokers aged 18–40 years who had no systemic disease were included in the study. In the included patients, there were no inflammatory symptoms in the oral cavity, ITM-induced trismus, pain or swelling, or advanced periodontal disease during surgery. Pregnancy, use of any medication in the 2 weeks before the procedure, presence of allergy to the medications to be used in the study (articaine, DXN, amoxicillin, paracetamol), lack of continuity in the follow-up process, and use of medications other than the prescribed medications were the exclusion criteria for the study. In addition, patients who experienced a complication during the tooth extraction procedure and whose operation time was longer than 30 min and patients who required second anesthesia during the procedure were not included in the study. In addition, complications such as infection, alveolitis, and bleeding in the postoperative period were also determined as exclusion criteria. All patients were informed about the study and signed an informed consent form.

### Study design and sampling

The patients were randomly divided into 3 groups. Randomization of the groups was done by drawing lots. Non-probability sequential sampling technique was used to select the required sample. The first group was the intravenous dexamethasone group (IVD), the second group was the perineural dexamethasone group (PND), and the third group was the control group. IANB (1.8 ml of articaine hydrochloride + 1 ml of saline) was performed 1 h after IV-DXN (4 mg/ml DXN) was administered to the IVD group (15 patients). PN-DXN along with IANB (1.8 ml of articaine hydrochloride + 1 ml of 4 mg/ml DXN) was applied to the PND group (15 patients). Only IANB (1.8 ml of articaine hydrochloride + 1 ml of saline) was applied to the control group (15 patients). The patients were divided into groups by drawing lots. The patient assigned to a group was given a code by the surgeon (DIK) who would perform the surgery. Postprocedure observations and measurements were made by another maxillofacial surgeon (BÖ).

The duration of anesthesia was the primary outcome of the study. In patients who would underwent ITM extraction, the enjection time of IANB was noted. If anesthesia was successful, the surgical procedure was performed. Patients who completed the surgery were taken to a separate observation room, and the duration of anesthesia was determined. A vitalometer device (Coxomedical instrument co. Ltd, China) was used to determine the duration of anesthesia. The tests began 1 h after anesthesia, and the tests were repeated every 15 min. For measurement with a vitalometer, the teeth were isolated with cotton rolls, and the teeth were dried with a sponge. A vitality test was performed on the buccal surface of the molars by applying toothpaste to the probe of the vitalometer. In cases where one of the molar teeth was missing, only the existing tooth was measured. The vitality test indicated values between 0 (no output) and 64 (maximum output). During the tests, the time when the patient first started to feel sensitivity was recorded.

The secondary outcomes of the study were edema and pain. To evaluate swelling in patients who underwent surgery, tragus-pogonion, tragus-oral canthus and gonion-lateral canthus were measured. Then, a single swelling value was obtained by taking the arithmetic average of these measurements. A tape measure that followed the contours of the face was used for measurement. For pain, the patients’ pain that day was evaluated with the 10-unit (0 no pain − 10 unbearable pain) visual analog scale (VAS) on the first, third and seventh days. Measurements were repeated on the first, third and seventh days.

### Surgical procedure

All patient procedures were performed by the same surgeon in the same room under the same conditions. After the patients were taken to the room for the procedure, extraoral asepsis was provided with 2% chlorhexidine gluconate solution. For intraoral asepsis, the patient was asked to rinse with 15 ml of 0.12% chlorhexidine gluconate gargle for 1 min.

IANB was applied directly using the anesthesia solution prepared according to the group of patients. After anesthesia was achieved, a sulcular incision was made in all patients, starting from the mesial aspect of the second molar tooth, using a No. 15 scalpel. Additionally, an alveolar incision was made from the distal of the second molar tooth to the vestibule side, and a full-thickness triangular flap was raised. The bone around the tooth was removed with a round bur under continuous saline irrigation. A physio dispenser set to 1200 rpm and 30 torque was used for bone reduction. After the tooth was extracted with the help of an elevator, the extraction socket was examined. If necessary, the dental follicle residues were removed, and the socket was irrigated with saline. After bleeding control was achieved, the flap was closed primarily with 3 − 0 silk suture in its original position. A small sponge was used to control the bleeding, and the postextraction instructions were explained to the patient. Nothing was said about applying cold compresses after the procedure. All patients were prescribed 1000 mg amoxicillin + clavulanic acid and 500 mg paracetamol twice a day after the procedure. It was said that the prescribed medications should be used for 5 days.

### Statistical analysis

All the statistical analyses were performed with the help of the statistical software language R version 4.1.2 (The R Foundation for Statistical Computing, Vienna, Austria; https://www.r-project.org). Before the analysis, the normality of the data was determined with the help of Shapiro‒Wilk’s normality test and Q‒Q tests. The assumption of sphericity was checked with the Mauchly test. The findings of the numerical variables in the study are presented as the mean ± standard deviation, and categorical variables are presented as frequencies (n) and percentages. Whether there was a statistically significant difference between the pain and edema levels of the IVD and PND groups on the 1st, 3rd and 7th days was evaluated by repeated measures analysis of variance and then by the Bonferroni corrected t test. If the assumption of sphericity was not met, multiple comparisons were made with the Bonferroni-corrected t test after Greenhouse‒Geisser corrected repeated measures variance analysis. In addition, whether there was a statistically significant difference between the pain and edema levels of the control, IVD and PND groups at each time point was evaluated by one-way analysis of variance (ANOVA). Then, the data were evaluated using Welch’s F test with Tukey’s HSD multiple comparisons and then Games-Howell multiple comparisons test.

## Results

A total of 45 patients, 25 (55.6%) women and 20 (44.4%) men, with an average age of 22.95 ± 2.70 years (age range 18–31), were included in the study. A comparison of the demographic characteristics and anesthesia durations of the control, IVD and PND groups is given in Table 1. According to the findings, the average age of the patients in the control, IVD and PND groups and sex distribution were similar. Additionally, the duration of anesthesia in the IVD group was significantly greater than that in the control group.

**Table 1:** Comparison of demographics and anesthesia durations of groups regarding different DXN administration routes

	Control (*n* = 15)	IVD (*n* = 15)	PND (*n* = 15)	*p* Value
Age	23.40 ± 3.41	22.60 ± 2.02	22.86 ± 2.61	.721^1^
Sex (M/F)	7 (46.7)/8 (53.3)	6 (40)/9 (60)	7 (46.7)/8 (53.3)	> .999^2^
Anesthesia duration	189 ± 49.36^a^	256 ± 79.46^b^	231.33 ± 85.03	**.049** ^**1**^

^1^One-way Analysis of Variance

^2^Pearson’s chi-square test

IVD: intravenous dexamethasone group

PND: Perineural dexamethasone group

Results with statistically significant differences are indicated in bold

The data are presented as the means ± standard deviations or frequencies (n) and percentages (%)

Comparisons of pain levels between the control, IVD and PND groups and on the 1st, 3rd and 7th days are given in Table 2. According to the findings, the pain level in the control group was significantly lower on the 7th day than on the 3rd day. Additionally, in the IVD group, the pain level was significantly lower on the 7th day than on the 3rd day. On the other hand, no significant change in pain level over time was observed in the PND group (*p* > 0.05). Additionally, when the pain levels in the groups were examined, the pain level in the IVD group was significantly lower on the 3rd day than that in the PND group. In addition, the pain level on the 7th day was significantly greater in the PND group than in the control group and the IVD group. On the other hand, there was no significant difference between the pain levels of the control, IVD and PND groups on day 1 (*p* > 0.05).

**Table 2:** Comparison of pain levels between the control, IVD and PND groups and on the 1st, 3rd and 7th days

Variables	Pain (VAS)							
	1st day (*n* = 45)	3rd day (*n* = 45)	7th day (*n* = 45)	*p* value^b^	1–3	1–7		3–7
Control	2.53 ± 1.80	3.20 ± 1.93	1.40 ± 1.18	**.002** ^**1**^	0.250	0.106		**0.002**
IVD	2.60 ± 2.02	2.13 ± 1.84	0.93 ± 1.38	**.011** ^**1**^	0.561	0.065		**0.035**
PND	3.33 ± 1.39	4.33 ± 2.05	3.26 ± 2.12	.251^1^	0.389	0.995		0.304
*p* value^a^	.395^2^	**.013** ^**2**^	**.006** ^**3**^					
Control-IVD	0.994	0.302	0.588					
Control-PND	0.436	0.260	**0.018**					
IVD-PND	0.496	**0.010**	**0.004**					

^1^Repeated measures analysis of variance assuming sphericity

^2^One-way Analysis of Variance

^3^Welch’s F test

*P* value^a^ comparison of pain levels between groups on each measurement day

*P* value^b^: comparison of pain levels between measurement times in each group

IVD: intravenous dexamethasone group

PND: Perineural dexamethasone group

Results with statistically significant differences are indicated in bold

The data are presented as the means ± standard deviations or frequencies (n) and percentages (%)

A comparison of edema levels between the control, IVD and PND groups and on the 1st, 3rd and 7th days is given in Table 3. According to the findings, the edema level in the control group was significantly lower on the 7th day than on the 1st and 3rd days. In the IVD group, the edema level was significantly lower on the 7th day than on the 1st and 3rd days. Additionally, in the PND group, the edema level was significantly lower on the 7th day than on the 1st and 3rd days.

**Table 3:** Comparison of edema levels between control, IVD and PND groups and on the 1st, 3rd and 7th days

Variables	Edema								
	1st day (*n* = 45)	3rd day (*n* = 45)	7th day (*n* = 45)		*p* value^b^	1–3	1–7		3–7
Control	11.24 ± 1.10	11.43 ± 1.33	10.92 ± 1		**.039** ^**2**^	0.068	**0.038**		**0.040**
IVD	10.67 ± 0.86	10.71 ± 1.03	10.04 ± 0.87		**< .001** ^**1**^	0.913	**< 0.001**		**< 0.001**
PND	10.52 ± 0.61	10.16 ± 0.63	10.01 ± 0.75		**< .001** ^**1**^	0.447	**0.003**		**0.026**
*p* value^a^	.076^3^	**.048** ^**4**^	**.010** ^**3**^						
Control-IVD	0.197	0.248	**0.024**						
Control-PND	0.080	**0.041**	**0.020**						
IVD-PND	0.889	0.638	0.997						

^1^Repeated measures analysis of variance with the assumption of sphericity

^2^Repeated measures analysis of variance with Greenhouse‒Geisser correction

^3^One-way Analysis of Variance

^4^Welch’s F test

*P* value^a^: comparison of edema levels between groups on each measurement day

*P* value^b^: comparison of edema levels between measurement times in each group

IVD: intravenous dexamethasone group

PND: Perineural dexamethasone group

Results with statistically significant differences are indicated in bold

The data are presented as the means ± standard deviations or frequencies (n) and percentages (%)

## Discussion

In this study, it was aimed to observe the increase in anesthesia duration after PN and IV administration of DXN in addition to IANB using articaine. In addition, it was compared which application method would be more effective in cases of pain and swelling that reduce the comfort of patients after ITM extraction.

Two studies evaluated the effect of PN administration via DXN on anesthesia duration during ITM extraction. One of these studies evaluated the duration of activity of IANB by adding DXN to lignocaine [9]. The other evaluated the effectiveness of DXN added to ropivacaine or bupivacaine on anesthesia duration [6]. This study is important because it is the first study in which DXN was used together with articaine in studies evaluating anesthesia duration in PN applications. In addition, the swelling and pain that occur in patients after IV administration of DXN have been evaluated in many studies [11–15]. However, the duration of effectiveness of IANB was not evaluated in these studies. In this respect, this paper is important because it is the first study to evaluate the duration of the effectiveness of IANB in IV applications of DXN.

When anesthesia durations were observed, it is seen that anesthesia was completed earlier in the control group (189 ± 49.36 min) than in the IVD group (256 ± 79.46 min) and PND group (231.33 ± 85.03 min). These data appear to be similar to those of other studies evaluating anesthesia duration. In one of these studies, the effect of lignocaine on anesthesia duration was measured [9]. However, in this study, a comparison was made according to the time it took for the numbness to completely disappear as an outcome of the duration of anesthesia. However, it can be concluded that DXN increases the duration of anesthesia when applied together with lignocaine. On the other hand, the duration of complete disappearance of numbness and the duration of the operation are different descriptions. For this reason, in this study, it was evaluated the time of anesthesia to the time of wear off pulpal anesthesia by using a vitalometer. In their study, Stojanovic et al. measured the effect of ropivacaine and dexamethasone on the duration of anesthesia when used together [6]. In this study, a comparison was made according to the time during which the numbness disappeared completely. However, in both of these studies, it was proven that DXN prolongs the duration of anesthesia in PN applications. In this study, it was observed that the duration of action of the articain and therefore the procedure time increased in both the PND and IVD groups. Moreover, the procedure time was significantly longer in the IVD group. Both methods prolong the duration of anesthesia in patients in whom DXN is administered via PN or IV and in patients who undergo supraclavicular brachial plexus block [16]. In addition, in this study, it was proven that the IVD group provided longer anesthesia compared to the PND group, although the difference was not significant. In this sense, the results regarding anesthesia duration are similar to this study.

Edema, which is another outcome of the study, decreased with the effect of DXN. Here, a distinctive effect was observed on the 3rd day in the PND group. However, on the 7th day, there was less swelling in both the IVD group and the PND group than in the control group. While the measurements of the distance between tragus-pogonion, tragus-mouth canthus and gonion-lateral canthus were generally compared statistically in the evaluation of swelling after surgical removal of the ITMs, it has been preferred to compare the averages of these values in this study. The reason for this was that the analyses performed in the study could be read more simply and understandably. For this reason, it was decided to evaluate the average of the three measurements preferred by Priyanga et al. in their study [17]. This results are not surprising, as it is known that DXN reduces the edema that occurs after surgical extraction by reducing the release of prostaglandins, lymphokines, bradykinin and serotonin from the injured tissue [18]. 

An unexpected result was encountered in pain measurements. Pain was significantly greater in the PND group than in the control and IVD groups on the 3rd and 7th days. There may be two reasons for this. First, DXN does not have the expected effect on pain in PN applications. In a study where IANB was applied by adding lignocaine DXN or adrenaline and pain was measured, it was observed that pain increased against DXN at the end of the first 27 h [9]. The second possibility may depend on the local anesthetic solution used, articaine. The addition of DXN to Articain during local application may cause more pain in the postoperative period, contrary to what is expected as a result of PN area applications. In their study, Atalay et al. compared PN administration of different doses of DXN combined with articaine with the control group [19]. Although no measurements were made regarding pain, they showed that the number of analgesics taken after the procedure was similar in all three groups. These results make sense of the increased pain after PN application via the DXN with articaine, which it was obtained in this study.

The most important limitations of this study are the sample size and the similar age distribution of the participants. The reason for the small sample size is that was expanded the exclusion criteria by reducing variables such as systemic disease, cigarette consumption, and tooth impaction classification in the study. An attempt was made to reach a clearer conclusion about the drugs administered by reducing the number of variables. Since the age distributions are close to each other, the indication for ITM extraction is generally in the 3rd decade. One of the most important reasons why DXN is a drug frequently preferred by surgeons in clinical practice is the reduction in postoperative sequelae. However, this study showed that it may also contribute to the duration of the surgical procedure. To determine the optimal application route and dose of DXN, studies comparing different doses and different application routes are needed.

## Conclusions

PN applications of 4 mg of DXN combined with 1.8 ml of articaine and 4 mg of DXN administered IV 1 h before the procedure prolonged the IANB time and therefore the ITM surgery procedure time. Additionally, PN and IV DXN applications helped reduce postoperative edema in patients. However, it has been observed that pain increases in PN applications of DXN with articaine. Studies measuring pain in PN applications of DXN will shed light on this issue in the future.

## Data Availability

The data sets used and/or analysed during the current study are available from the corresponding author on reasonable request.

## Abbreviations

ITM: Impacted third molar
DXN: Dexamethasone
IV: Intravenous
PN: Perineural
IANB: Inferior alveolar nerve block
IVD: Intravenous dexamethasone group
PND: Perineural dexamethasone group
VAS: Visual analog scale
